# The Group Nurturance Inventory — initial psychometric evaluation using Rasch and factor analysis

**DOI:** 10.1186/s12889-021-11474-5

**Published:** 2021-07-26

**Authors:** Magnus Johansson, Anthony Biglan

**Affiliations:** 1 Department of Behavioural Science, Oslo Metropolitan University, P.O. Box 4, St. Olavs plass, NO-0130 Oslo, Norway; 2 Oregon Research Institute, Eugene, USA

**Keywords:** Nurturing environments, Rasch, Psychometrics, Organizations, Measurement, Open science

## Abstract

**Background:**

This paper describes the development and psychometric evaluation of a behavioral assessment instrument primarily intended for use with workgroups in any type of organization. The instrument was developed based on the Nurturing Environments framework which describes four domains important for health, well-being, and productivity; minimizing toxic social interactions, teaching and reinforcing prosocial behaviors, limiting opportunities for problem behaviors, and promoting psychological flexibility. The instrument is freely available to use and adapt under a CC-BY license and intended as a tool that is easy for any group to use and interpret to identify key behaviors to improve their psychosocial work environment.

**Methods:**

Questionnaire data of perceived frequency of behaviors relevant to nurturance were collected from nine different organizations in Sweden. Data were analyzed using confirmatory factor analysis, Rasch analysis, and correlations to investigate relationships with relevant workplace measures.

**Results:**

The results indicate that the 23-item instrument is usefully divided in two factors, which can be described as risk and protective factors. Toxic social behaviors make up the risk factor, while the protective factor includes prosocial behavior, behaviors that limit problems, and psychological flexibility. Rasch analysis showed that the response categories work as intended for all items, item fit is satisfactory, and there was no significant differential item functioning across age or gender. Targeting indicates that measurement precision is skewed towards lower levels of both factors, while item thresholds are distributed over the range of participant abilities, particularly for the protective factor. A Rasch score table is available for ordinal to interval data transformation.

**Conclusions:**

This initial analysis shows promising results, while more data is needed to investigate group-level measurement properties and validation against concrete longitudinal outcomes. We provide recommendations for how to work in practice with a group based on their assessment data, and how to optimize the measurement precision further. By using a two-dimensional assessment with ratings of both frequency and perceived importance of behaviors the instrument can help facilitate a participatory group development process. The Group Nurturance Inventory is freely available to use and adapt for both commercial and non-commercial use and could help promote transparent assessment practices in organizational and group development.

**Supplementary Information:**

The online version contains supplementary material available at 10.1186/s12889-021-11474-5.

## Introduction

This paper describes the development of a measure of nurturing social behavior in work environments. It is intended for group-level assessments at workplaces. The measure is freely available and designed to be easy to use and helpful in pinpointing targets of behavior change that could improve key social interactions in workplaces so that groups can evolve without depending on external actors. This paper describes and analyzes the Swedish version.

Social interaction at work is important for understanding how people are affected by their work environment, and how well-being, health, and productivity can be influenced by social factors. The importance of social interactions in the work environment has been investigated over decades of research, in terms of well-being [1–4], stress [5, 6], burnout [7–11], mental health [12–14], physical health [15–19], sick-leave [20, 21], as well as productivity [14, 22–25] and profitability [26, 27]. Thus, being able to assess and improve key social aspects of the work environment can potentially be beneficial to all these areas.

Identifying and intervening on risk and protective factors to create nurturing environments that promote a healthy developmental trajectory for children and youth are at the core of prevention science. We argue that the same concept can be useful for assessment and intervention aimed at adults and workplaces. Research in prevention science proposes that nurturing environments have four core domains [28, 29]: minimizing toxic social interactions; teaching and reinforcing prosocial behaviors; limiting opportunities for and influences of problem behaviors; and promoting psychological flexibility. To explore the assumption that the nurturing environments framework is useful for evolving nurturance in groups and organizations, we set out to develop a measure of nurturance in workplaces, focusing on group-level assessment.

Behavior analysts have suggested that the aggregate product of groups is a function of the interlocking behaviors of group members [30–32]. One of the key features of such interlocking behavior is the extent to which group members support and reinforce each other’s contributions to the group’s product and minimize coercive behavior. We believe that the four features of the nurturing environments framework could be useful in identifying and assessing the key social aspects of groups’ interlocking behaviors. Thus, we propose that it could be useful to create a reliable and valid measure of nurturance in workgroups.

Is there a need for another assessment instrument aimed at workgroups? Several group assessment tools have been developed and are widely used in practice [33–36], but most are limited by copyright restrictions and certification systems. One of the reasons to develop this instrument was to encourage an open-source approach to organizational assessment and change that can be further adapted, and also utilized commercially if desired. The proposed measure is also different from most workplace measures in that it assesses both frequency and preferences of social interaction behaviors.

The job demand, control, and social support model [37] and the effort-reward imbalance model [19] are often referred to as the dominant theories in psychosocial work environments [12, 13, 38–40]. These models typically assess the social aspects of work environments using questionnaires focused on individual experiences. Examples of items from rating scales for these models are “My colleagues are there for me”, “There is a good spirit of unity” [41], “I receive the respect I deserve from my superior or a respective relevant person”, and “Over the past few years, my job has become more and more demanding” [42]. Subjective data about experiences is useful to understand how workers perceive the social environment at work. However, it is less likely to be useful when the ambition is to identify specific actions that could be taken to improve the quality of social interactions at work. A measure that focuses on specific observable behavior could be easily interpreted and used to guide change efforts, without the need for external actors. In line with this reasoning, it is likely that assessment feedback to respondents would be more helpful if provided for each item rather than only sum scores so that the behavioral specificity is retained.

When the development of this assessment instrument started, it was conceived as a form listing behaviors to be used when conducting structured observations of social interaction behaviors in workgroups. However, since observational data is very resource-demanding to collect reliably, particularly in sufficient amounts for psychometric analysis, it was decided to first devise and test a self-rated version of the form, while keeping the focus on observable behaviors. The assessment instrument was named Group Nurturance Inventory (GNI).

While self-ratings have many inherent weaknesses, such as recency bias [43], it enables the collection of some types of data that cannot be directly observed. To make use of this, the GNI is used in two complementary ways: (a) to estimate the frequency of behaviors in a group, as assessed by members of the group; (b) to estimate how important each member perceives that these behaviors are, i.e. a preference assessment. We believe that taking group-level preferences into account improves the utility of the measure, which can help guide and motivate change work [44].

This paper focuses on analyses of individual-level data on the frequency rating part of the Swedish version of the assessment instrument, using confirmatory factor analysis as well as Rasch analysis, and also investigates relationships with other measures. While the intended use of this measure is primarily as a group-level assessment, the individual-level properties should be investigated before moving to group-level analysis [45, 46].

## Materials and methods

### Participants

**Fig. 1:**
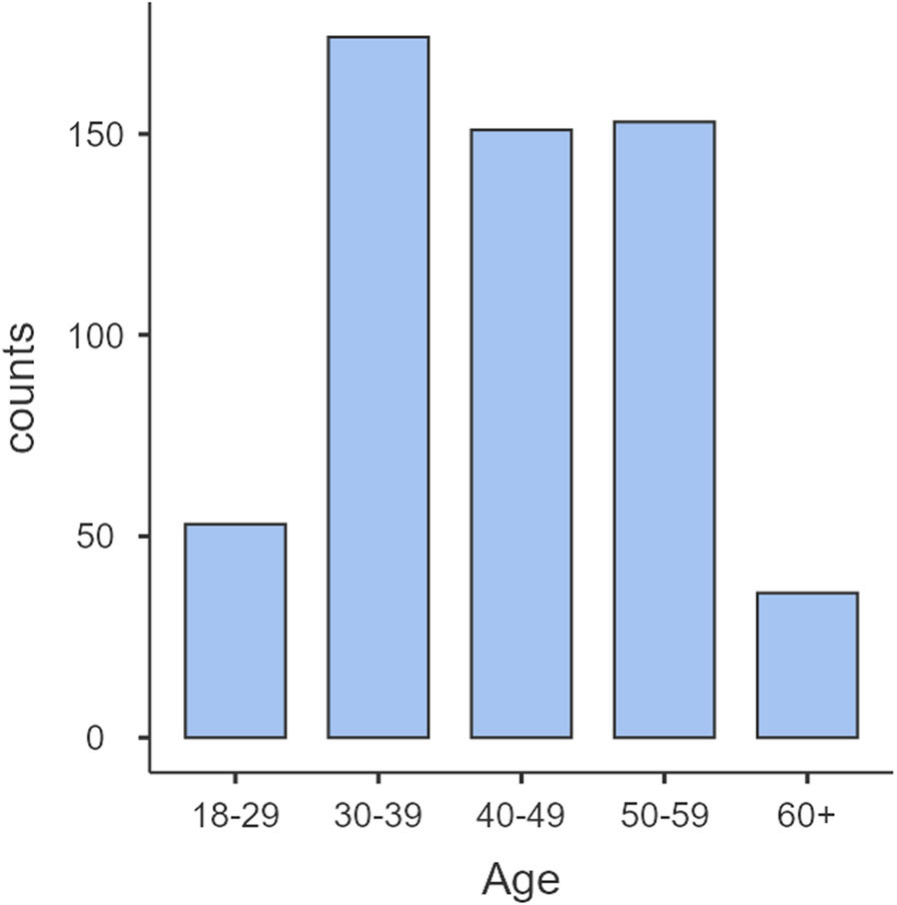
Age distribution of participants

The dataset consisted of 582 participants (27.4% female) from nine organizations in different sectors (forestry, infrastructure, banking, health care, construction,administrative authority, and fire services). While most participants represented a unit within a larger organization, one large organization had 13 units. Participant age was collected in decade intervals, with 40–49 being the median (see Fig. 1 for age distribution data).

### Design

This study involved four parts: item generation and pilot testing; internal validity and dimensionality by confirmatory factor analysis and Rasch analysis; and relationships with relevant workplace measures.

### Initial scale development

Using the four domains of the Nurturing Environments (NE) framework as described in the original paper by Biglan et al. [29], a focus group consisting of 10 management consultants (50% female, age range 31–55) contributed individual and collectively formed suggestions for overtly observable behaviors that would characterize each of the four NE domains in work-groups. The suggestions were analyzed, summarized, and structured into items, creating the first version of the form. Great care was taken to pinpoint the most important behaviors while keeping the form relatively brief. This resulted in varying levels of behavioral specificity amongst the items. While some items are highly specific, some “break the rules” of good practice in item construction by, for example, encompassing two behaviors. The aim was to strike a balance between utility, brevity, and good-enough item construction.

A smaller focus group, which included the authors of this paper and two of the original focus group members, provided feedback on the suggested items. An initial 19-item self-rated form was tested with a healthcare organization. Based on qualitative input, minor changes were made and four items were added. A 23-item form was first created in Swedish and later translated to English in accordance with the ISPOR guidelines [47]. A Norwegian translation is also available. Questionnaires are freely available, see Availability of data and materials.

While the first three domains of Nurturing Environments (toxic social behaviors, prosocial behaviors, and behaviors that limit or prevent problems) seem straightforward enough to identify overt behaviors, the fourth domain is more complex. Psychological flexibility (PF) is a key construct in Acceptance and Commitment Therapy [48], and there are several variants of self-rated PF measures, both for general use [49] and for specific target groups or contexts [50]. As the GNI aims to assess group-level PF, this is a new venue for PF measurement. Existing measures focus on how the individual deals with internal experiences (thoughts, feelings, sensations, etc), while also describing being able to take valued action, which can be an observable behavior. Assessing PF by focusing only on overt behaviors has to our knowledge not been attempted previously.

### Instruments

The main measure was the GNI-23 in its Swedish language version. Participants were asked: “At your workplace, how often do you, your colleagues or your manager...” followed by the 23 items, each with the four response categories: “Never/almost never, Seldom, Fairly often, Very often.” Examples of items include “create opportunities for follow-up/feedback,” and “interrupt the person speaking.” See Table 1 in the results section for all items, and Availability of data and materials for links to the questionnaire and available translations.

**Table 1:** Confirmatory Factor Analyses of the GNI-23

Model	χ^2^	RMSEA	CFI	TLI	SRMR
4-factor	565.75, *df* = 224, *p* < .000	.051 (90% *CI* = .046–.056)	.924	.914	.068
4-factor^a^	424.00, *df =* 224, *p* < .000	.039 (90% *CI* = .033–.045)	.956	.950	.057
3-factor^b^	426.40, *df* = 227, *p* < .000	.039 (90% *CI* = .033–.044)	.956	.951	.058
2-factor^c^	460.27, *df* = 229, *p* < .000	.042 (90% *CI* = .036–.047)	.956	.951	.068

^a^Item 21 moved from Psychological Flexibility (PF) to Non-toxic.

^b^Limit Problems and PF merged to one factor.

^c^PF/Limit problems merged with Prosocial.

*CI* Confidence Interval, *RMSEA* Root Mean Square Error of Approximation, *SRMR* Standardized Root Mean square Residual, *CFI* Bentler’s Comparative Fit Index, *TLI* Tucker-Lewis Index

Six items (numbers 1–6) were a priori assumed to indicate the “Toxic social behavior” domain, and a high rating is expected to be undesirable. Item 21 is also assumed to be undesirable. Items 7–14 were designated to the domain of “Prosocial behaviors”, 15–18 to “Limit problem behaviors”, and 19–23 to the “Psychological flexibility” domain. To easily be able to compare the scores, the items describing undesirable behaviors (1–6 and 21) are reverse scored to consistently have high scores as desirable. The reverse scoring means that the domain “Toxic social behaviors” is renamed “Non-toxic” in the results section, to simplify interpretation.

The self-rated perceived frequency of the 23 items is the data collected using the instrument analyzed in this paper. When collecting data, participants were also asked about their perceived importance of each item. This was done by asking “How important is it that you, your colleagues and manager are good at...”, with each item having four response options: “Not important at all, Fairly unimportant, Fairly important, Very important.” For the undesired behavior items, the word “not” was added at the start of the item in the importance rating, resulting in questions such as “How important is it to NOT interrupt the person speaking?”

Based on the aggregated group assessment of frequency and importance, a difference score can be calculated. This is of course a rough estimation, since the raw score ratings are not interval level data. However, the difference score intuitively seems like a good indicator for targets of change, and could be pragmatically useful. For instance, if the members of a group rate the frequency of “asks how work tasks are proceeding” as low, while indicating a high grade of importance for the same item, this could indicate a discrepancy between the perceived situation and the desired one, and the group would likely benefit from increasing the frequency of this behavior.

Seven workplace instruments were chosen to investigate the convergent and discriminant validity of the GNI by analyzing relationships with other variables. The Demand, Control, Social Support, and Effort/Reward Imbalance models were both relevant to include because of the large amount of existing research and their connections to many relevant outcomes, for instance health and productivity. Other variables of interest included perceived stress, interpersonal trust, job satisfaction, negative acts, enjoyment of work, and meaningfulness of work.

The Work Acceptance and Action Questionnaire (WAAQ) [51], is a work-specific measure of Psychological Flexibility consisting of seven items rated on a 1–7 point scale (“Never true” to “Always true”). This measure is of particular interest since it represents a domain of the Nurturing Environments framework. The Swedish translation of WAAQ has previously been analyzed using principal component analysis [52], but the WAAQ has not previously been subject to confirmatory factor analysis (CFA) and will be more extensively explored in the results section.

The 10-item version of the Perceived Stress Scale [53, 54] was used, with Cronbach’s α in the current sample at .83. Data from a large Swedish population sample [55] showed a mean score of 14.0 (*SD* = 6.34), while the sample in this study had a mean score of 12.8 (Median 13.0, *SD* = 5.45, Range 0–31).

The Demands, Control, and Social support Questionnaire consists of 17 items with three subscales [41, 56]. Cronbach’s *α* in the current sample was .74 for the Demands subscale, .52 for Control, and .85 for Social Support. The low Cronbach’s α for the Control subscale was in line with previous findings in Swedish samples [57], where it was split into Skill Discretion (4 items) and Decision Authority (2 items). However, that solution did not result in a satisfactory model fit with the current dataset, with both Cronbach’s α and CFA indicating problems. Thus, the Control subscale was deemed not suitable for use in this dataset and will not be included in the analyses. Following the recommendations by Chungkam et al. [57], item 2 in the Demands subscale was removed, which resulted in improved CFA model fit, while Cronbach’s α decreased to .69.

The Effort/Reward Imbalance was measured with the 10-item version ERI-S [42, 58], which contains 7 items for the Reward factor (divided into subfactors of Esteem, Security, and Promotion) and 3 items for the Effort factor. Cronbach’s α for the Effort factor was .75, and for the Reward factor α = .77. An effort/reward ratio is calculated based on the effort and reward ordinal sum scores. Interpersonal trust is another important indicator of group functioning [59–61] and psychological safety [62]. In this study, trust was measured using six items from the Interpersonal Trust scale created by Cook and Wall [63], with Cronbach’s α = .84.

Two single-item questions were used to measure work satisfaction and work meaningfulness on a 1–7 scale. Previous research has shown that for this particular purpose, single-item questions can often be sufficient [64–66]. The Short Negative Acts Questionnaire [67] was used as a measure of bullying at work, using 9 items with 5 response options each. Cronbach’s α was .85. Three of the organizations contributing data also answered single-item questions about comprehensibility and how well the GNI items represented behaviors relevant to their work environment (*N* = 79, response scale 1–7).

### Statistical procedures

Data were collected using the survey tool Nettskjema.no and recoded into numerics using Rstudio 1.2.5042 [68, 69] with package “car” version 3.0–7 [70]. Kaiser-Meyer-Olkin value, Bartlett’s sphericity test, and Cronbach’s α statistics were calculated using software from The Jamovi Project, version 1.6.23 [71]. Confirmatory factor analyses and structural equation models with correlational analyses were done using Mplus 8.4 [72]. Rasch analyses were conducted using the RUMM 2030 software [73] and Winsteps software 4.7.1 [74].

Data had no missing values, although not all groups were asked to fill out all questionnaires, meaning that the number of participants in the correlation analyses will differ between instruments. As a consequence, some correlation analyses have less statistical power and might be less generalizable since fewer organizations are represented, for the Short Negative Acts Questionnaire (only fire services), Interpersonal Trust (infrastructure and fire services), and Effort/Reward Imbalance (administrative authority, banking, and health care). For the GNI, items 1–6 and 21 were reverse scored since they describe behaviors assumed to be undesirable. This was done to make all items have the same direction, with a higher score assumed to be desirable.

Model fit is assessed by multiple tests and fit indices [75]. Chi-square should be non-significant (*p* > .05), but it is not always a reliable indicator since it is sensitive to sample size and non-normally distributed data. The Standardized Root Mean square Residual (SRMR) is also reported, as well as the Root Mean Square Error of Approximation (RMSEA), Bentler’s Comparative Fit Index (CFI), and the Tucker-Lewis Index (TLI). Hu and Bentler [76] suggest that values below .08 for SRMR and .06 for RMSEA are considered a good fit, while CFI and TLI should be .90 or above. Since the GNI-23 uses four ordered response categories, a robust weighted least squares estimator using a diagonal weight matrix (WLSMV in M*plus*) was used for factor analyses [77]. Since participants belonged to multiple groups the clustered nature of the data [78] was taken into account and standard errors were adjusted by using “type = complex” and “cluster = org” specifications in M*plus*. For the correlation analysis, the maximum likelihood estimator was used with Rasch transformed interval scores for GNI factors and bootstrapping to estimate confidence intervals. Factor loadings are reported in their standardized form.

Rasch measurement theory [79, 80] is a mathematical measurement model based on the assumption that the probability of a person’s response to a questionnaire item is a “logistic function of the relative distance between the item location and the respondent location on a linear scale” [81 p.1358]. In other words, a person’s overall score on a scale (person location) consisting of multiple items should indicate the probability of responses to the scale’s items in a systematic way, based on their difficulty (item location). The Rasch analyses in this study were primarily focused on the aspects of psychometric assessment that Rasch measurement theory most clearly contributes beyond classical test theory, which included thresholds of response categories, item and person fit and location, differential item functioning (DIF), local independence, targeting, and person separation. DIF entails the investigation of item bias related to demographical variables (sex and age in this sample) to assess measurement invariance, which can be either uniform or non-uniform across class intervals [82]. We utilized the RUMM 2030 function for analyzing DIF, which includes analysis of variance (ANOVA) of item residuals and visual inspection of item characteristic curves [83]. As response category thresholds were expected to be approximately equal across the items, we used Andrich’s Rating Scale Model for polytomous data. The dataset is freely available, see Availability of data and materials. When assessing item fit, items should not have significant χ2 values or fit residuals beyond +/− 2.5 and the person separation index should be over .85 for individual use and .70 for group-level use based on the RUMM2030 output [81]. The Winsteps item mean square infit/outfit statistics should be within the 0.7 to 1.3 range, while correlated residuals, indicating issues with local dependencies, should be below .30 [84].

## Results

### Confirmatory factor analysis

Kaiser-Meyer-Olkin overall value for sample 1 was 0.860 (range 0.810–0.926), and Bartlett’s Test of Sphericity was significant (χ^2^ = 1847, df = 253, *p* < .001), indicating adequate sampling for factor analysis [85]. A confirmatory factor analysis (CFA) was conducted with the a priori defined model described earlier. While the 4-factor model indicated acceptable fit statistics (χ2 (224) = 565.748, *p* < .000, CFI = .924, TLI = .914, SRMR = .068, RMSEA = .051 (90% CI = .046–.056)), modification indices showed a χ2 of 129.589 for item 21 loading on the Non-toxic factor. A model with item 21 moved from Psychological Flexibility (PF) to Non-toxic resulted in improved fit (χ2 (224) = 424.000, *p* < .000, CFI = .956, TLI = .950, SRMR = .057, RMSEA = .039 (90% CI = .033–.045)), and meant that all questions that a priori were assumed to be undesirable now belong to the same factor.

However, the three factors with items describing desirable behaviors showed very high intercorrelations (Prosocial with PF *r* = .76, Limit Problems with Prosocial *r* = .82, and PF with Limit Problems *r* = .96). Merging PF and Limit problems to one factor resulted in a 3-factor model with almost identical model fit compared to the 4-factor model (χ2 (227) = 426.399, *p* < .000, CFI = .956, TLI = .951, SRMR = .058, RMSEA = .039 (90% CI = .033–.044)). Correlations between Prosocial and the merged factor for PF/Limit Problems remained high at *r* = .80. We specified a 2-factor model, merging Prosocial with PF/Limit Problems, which was found to also have good fit (χ2 (229) = 460.272, p < .000, CFI = .956, TLI = .951, SRMR = .068, RMSEA = .042 (90% CI = .036–.047)). A summary of the CFA model fit statistics is provided in Table 1. Cronbach’s α for the two domains was calculated at .80 for Non-toxic and at .88 for the domain created by merging Prosocial, Limit Problems and PF. Standardized factor loadings for the 2-factor model are detailed in Table 2.

**Table 2:** 2-factor model with standardized factor loadings

Item	Non-toxic	Prosocial/Limit Problems/ Psychological Flexibility
1. Interrupt the person speaking	0.578	
2. Look away, or at another person than the one speaking	0.641	
3. Harshly criticize or blame someone	0.779	
4. Use discriminatory language/jokes, or laugh at such	0.654	
5. Respond defensively in discussions	0.746	
6. Say that something is important, but act as if it is not	0.719	
7. Ask about or validate others’ needs/feelings/state		0.554
8. Ask how work task are proceeding		0.724
9. Offer help or ask for help		0.737
10. Invite others into conversation or socializing		0.672
11. Listen actively to the person speaking		0.701
12. Encourage and reinforce others’ behaviors and achievements		0.764
13. Express opinions in a constructive way		0.641
14. Talk about how people behave (instead of their traits or attitudes)		0.462
15. Remind others about values/rules/policies in close proximity to an activity where they apply		0.606
16. Create opportunities for follow-up/feedback		0.709
17. Discourage behaviors that are not ok		0.591
18. Deal with potential problems early on		0.693
19. Make decisions aligned with values/policy even when it might lead to short term losses or problems		0.596
20. Ask for dissenting opinions and listen to them		0.639
21. Speak impulsively, without considering other perspectives	0.719	
22. Summarize and confirm others’ arguments before own thoughts are expressed		0.472
23. Talk about shared values		0.713

### Rasch analysis

Rasch analysis of dimensionality utilizes principal component analysis and correlations of item residuals. Entering all 23 items into the analysis showed two separate item clusters, one of them being the Non-toxic factor, and the other consisting of the remaining items, identical to the 2-factor CFA model. Running separate Rasch analyses for these two clusters, item-trait interactions were found to be non-significant, indicating unidimensionality for each factor. Item fit was satisfactory for all items in both factors, with no items having significant χ^2^ values or fit residuals beyond +/− 2.5, and mean square infit/outfit statistics were all within the 0.7 to 1.3 range. The person separation index (PSI) was at .78 for Non-toxic and at .87 for the factor consisting of all remaining items. See Table 3 for a summary of Rasch statistics.

**Table 3:** Rasch analysis summary statistics

Domain/model	Item-trait interaction χ^2^	Person location mean (*SD*)	Person residual mean (*SD*)	PSI	Item difficulty range
Non-toxic	52.54, *df =* 56, *p* = 0.26	1.18 (1.62)	−0.55 (1.28)	.78	−0.43 to 0.90
Prosocial, PF & Limit Problems	139.91, *df* = 128, *p* = 0.22	0.82 (1.26)	−0.43 (1.50)	.87	−1.66 to 1.33

*PSI* person separation index, *SD* Standard Deviation

**Fig. 2:**
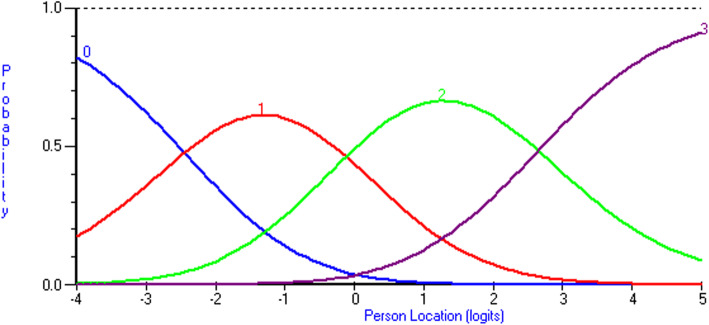
Probability of response categories for item 7 relative to person location

There were no disordered thresholds, indicating that the respondents reliably differentiated among the four response categories for all items. To illustrate the response category thresholds, Fig. 2 shows the probability of response categories for item 7 on the Y axis, with the person location on the X-axis. Thresholds are located at the points where two lines intersect.

**Fig. 3:**
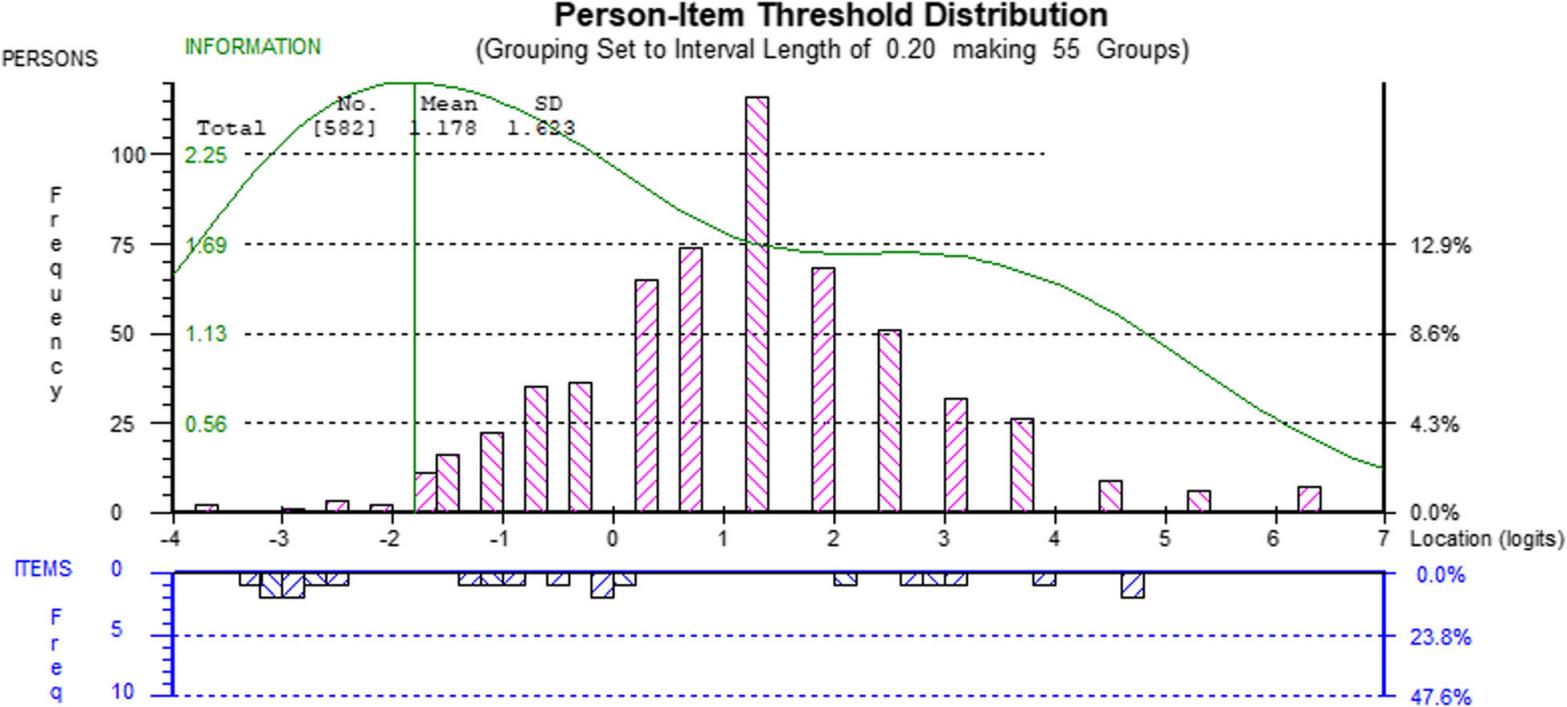
Person-Item Threshold distribution for the Non-toxic subscale

**Fig. 4:**
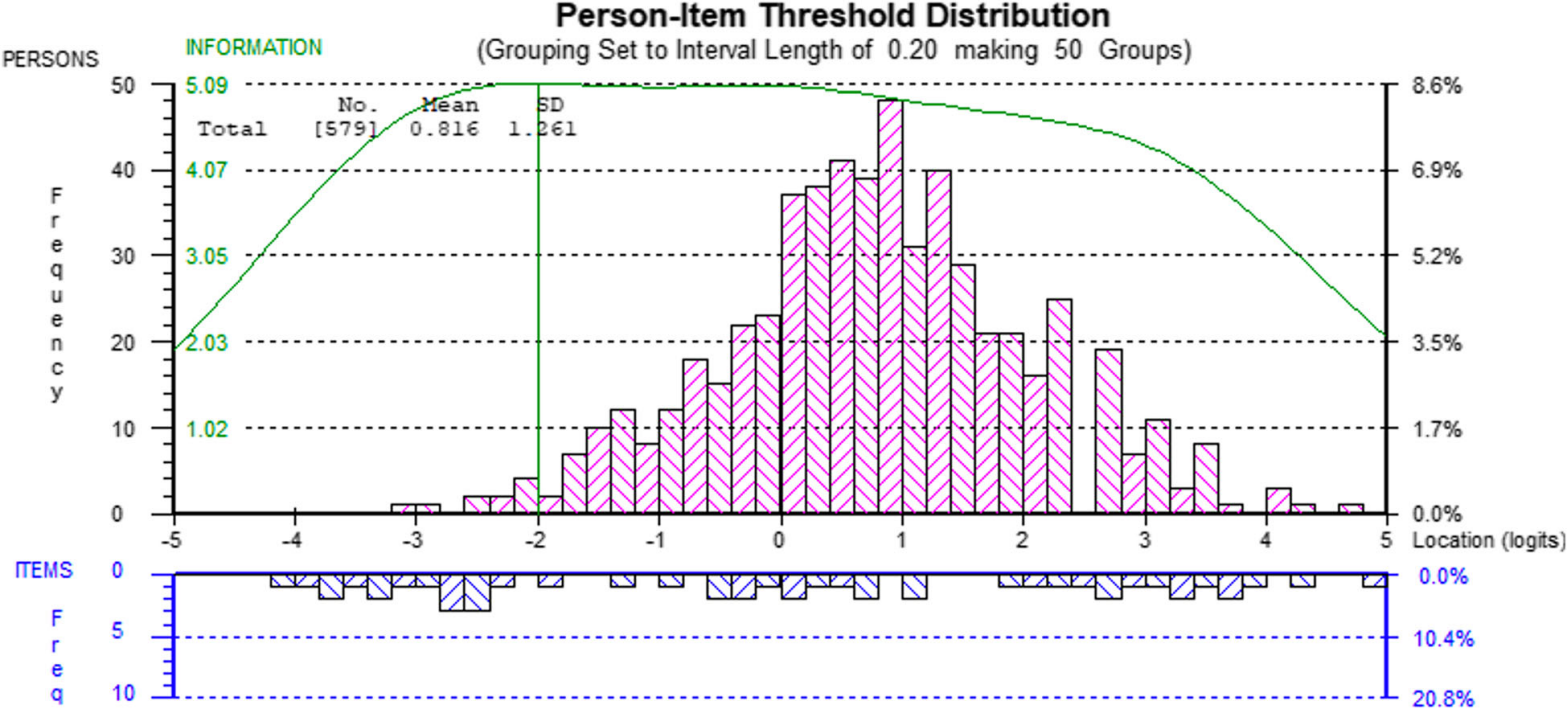
Person-Item Threshold distribution for the Prosocial and Limit Problems domains combined

Table 3 also shows the range of item difficulties (further detailed in the Additional file 1) and person location statistics for the two domains, where means and standard deviations should ideally be approximately 0 and 1, respectively. Figures 3 and 4 visualize item and person locations relative to each other by showing the person location distributions above the horizontal midline and the item response threshold distributions below the midline, both on the same logit scale. These figures also indicate the GNI’s targeting properties relative to the properties of the sample. There are notable gaps in item thresholds where there are persons for the Non-toxic domain, particularly at 0.5 to 2 logits. The green line in the figures describes optimal targeting, which peaks at the line approximately 2.5 logits below the person average in this sample. The prosocial/limit problems domain has more and wider spread item threshold locations compared to person locations. A more detailed visualization of item thresholds on the item level is available in the Additional file 1.

There was no significant differential item functioning for sex or age group (divided into decades) for any item, nor any local dependencies above the 0.30 level.

### Convergent and discriminant validity

230 participants had filled out the WAAQ, and a single-factor confirmatory factor analysis model showed acceptable but not optimal fit using the maximum likelihood estimator (χ2 (14) = 26.74, *p* = .021, CFI = .986, TLI = .979, SRMR = .025, RMSEA = .063 (90% CI = .024–.099)). Factor loadings ranged between .54 and .82 and Cronbach’s α was .90. The sample in the Swedish WAAQ paper by Holmberg et al. [52] had a mean WAAQ score of 33.6 (SD = 5.42), while the current sample had a mean score of 33.2 (Median = 33.0, SD = 6.91, Range 14–49). Similarly to what Holmberg et al. [52] found, removing item 2 improved Cronbach’s α by .005 and also resulted in a better model fit (χ2 (9) = 15.05, *p* = .09, CFI = .993, TLI = .988, SRMR = .018, RMSEA = .054 (90% CI = .000–.100)), notably improving the RMSEA index below the recommended threshold of .06 for good fit [76].

A Bonferroni correction for 18 comparisons set the level of statistical significance (adjusted from *p* = .05) to *p* = .003. Correlations in Table 4 were estimated using Rasch-converted interval level scores for the two GNI factors in structural equation models where the other constructs were specified as latent factors by their respective items, except for the two single-item questions and the Effort/Reward ratio. Not all participants had filled out all questionnaires, which is why the number of participants differs between different models.

**Table 4:** Correlations between Rasch-scored GNI-factors and other variables

Measure	Standardized correlation coefficients with 95% CI [LL UL]		Cronb. α	N
	Non-toxic	Prosocial/Limit Problems/PF		
Meaningful work	.25 [.17 .33]*	.31 [.23 .38]*	–	582
Enjoy work	.38 [.31 .45]*	.44 [.37 .50]*	–	582
Demands	−.16 [−.27–.05]	−.10 [−.22 .02]	.69	536
Social Support	.24 [.14 .33]*	.46 [.38, .55]*	.85	536
WAAQ	.07 [−.08 .24]	.22 [.06 .36]	.91	230
Perceived Stress	−.21 [−.32–.10]*	−.15 [−.25–.04]	.80	546
Effort/Reward ratio	−.30 [−.40–.20]*	−.34 [−.43–.24]*	–	336
Interpersonal Trust	.26 [.10 .45]	.46 [.25 .62]*	.84	316
SNAQ	−.47[−.59–.34]*	−.17[−.34 .01]	.85	291

*CI* Confidence Interval, *LL* Lower Limit, *UL* Upper Limit, *WAAQ* Work Acceptance and Action Questionnaire, *SNAQ* Short Negative Acts Questionnaire.

*p < .003

The single-item measures of item comprehension and perceived item relevance were filled out by a subsample in the early data collection (*N* = 79, scale 1–7), with comprehension ratings showing a mean score of 5.20 and *SD* = 1.17, and relevance ratings had a mean of 5.05 with *SD* = 1.28.

## Discussion

The issue of dimensionality was not as clearly connected to the Nurturing Environments framework as anticipated. While the Non-toxic factor was consistently shown to be unidimensional and sufficiently independent, the other factors had more complexity and stronger intercorrelations. The most parsimonious 2-factor model showed slightly worse fit indices, but still well within desired thresholds. From a prevention perspective, it could be argued that the Non-toxic domain describes risk factors, behaviors we want to have less of to lower the risk of undesired consequences, while the other items describe behaviors we are likely to benefit from having more of – protective factors [86, 87]. The 2-factor model was also supported by the Rasch analysis and can be used to aggregate GNI data and present an overall picture of the prevalence of nurturance in terms of risk and protective behaviors in a group.

At the same time, the intended practical use of the GNI relies on item level specifics to identify possible concrete behavior change targets, which makes the question of factor scores secondary. Any kind of score summarizing a factor will be less helpful in identifying what to change to make improvements. But at higher levels of organizations, where less detailed comparisons may be of greater interest, the model describing two factors may be sufficient and even desirable. For this purpose, we have used Rasch analysis to provide a table in the Additional file 1 that allows the transformation of ordinal sum scores to interval level scores with measurement uncertainties at each score level. Using the ordinal to interval conversion table is of course also highly recommended to use if one wants to utilize sum scores for any kind of statistical analysis.

We had expected the Psychological Flexibility (PF) factor to be difficult to pinpoint on the level of overt behavior, but did not expect the very strong correlation between the items making up PF and Limit Problems and the merging of the two factors. It is feasible that the same conditions that help limit problem behaviors also promote psychological flexibility. and also correlate to a large degree with prosocial behaviors that foster self-regulation. PF and self-regulation are arguably similar in that both describe the capacity to withhold from acting impulsively when facing unwanted sensations [88, 89].

The challenge of understanding the interpersonal aspects of psychological flexibility was recently highlighted [90], and it would seem our findings confirm that this is indeed difficult, perhaps one that necessitates a whole separate line of studies. Adding to the complexity, a recent review paper [91] criticizes the lack of coherence in defining PF in the applied research literature, and suggests the use of a newly developed, more idiographically flexible measure [92], which would be interesting and challenging to adapt to group level settings.

After creating the GNI measure and collecting the data used in this study, we were made aware of an effort to develop an organizational level measure of psychological flexibility [93], which found a correlation with individual-level psychological flexibility (measured with the WAAQ) similar to that of the GNI. Based on the organizational flexibility scale, we collaborated with Gascoyne to devise a measure intended to assess group-level flexibility. Unfortunately, the COVID-19 pandemic obstructed the data collection, resulting in insufficient data for analysis.

Correlations show that the relationships between the GNI factors and other workplace measures are along the expected lines, with medium to large but not overly large coefficients, which indicates that the GNI is covering similar but not identical facets of the work environment.

Based on the targeting analyses (Figs. 3 and 4), item thresholds are quite well distributed compared to person locations, while being somewhat skewed toward the lower part of the spectrum. This means that measurement precision is better for groups with lower levels of functioning on the GNI measure. To a high-functioning group or organization, some of the GNI behaviors may appear banal, but if key social interaction behaviors are failing, perhaps particularly toxic and prosocial behaviors, group members could probably benefit significantly from improving them. While it is a strength of this study that the data were collected from a range of real-world workplaces, the participants in this sample seem to be mostly well-functioning, as indicated by the Rasch analysis on targeting and more clearly by the reference levels on perceived stress that shows the sample to be below the expected Swedish average. Data from a population with a wider distribution of abilities, especially from groups with lower levels of functioning, would have strengthened the analysis.

As mentioned in the introduction, this paper has focused on the frequency ratings but also collected data on group members’ perceptions of the importance of the same interaction behaviors within their group. The importance ratings can be highly useful together with the frequency ratings and are probably particularly relevant at an initial assessment of a group. The importance rating can be seen as a form of preference assessment, not unlike values exercises that are often used at workplaces, but much more specific and actionable since we present overt behaviors to rate. Identifying potential discrepancies between the ratings of preferences and frequencies of behavior can have motivational functions for behavior change. For this to work properly, the feedback to the group should be on the individual item (behavioral) level, rather than just summarizing domain scores.

In our experience, most groups find it very difficult, even with guidance, to identify specific behaviors based on broader terms such as values, traits, or domains. Since the GNI prescribes specific behaviors, there could be a risk of undermining self-governance or self-determination. By asking about both perceived frequency and importance, we have a better foundation for retaining the participatory and collaborative part. The feedback session is important to achieve this effect, allowing for group discussions on every item, with extra attention given to those items that show the highest level of discrepancy between importance and frequency ratings. The “discrepancy score” is not a mathematically sound number since it is calculated from the raw ordinal data and created by deducting the group average frequency score from the group average importance score for each item, but it seems useful based on the feedback we have received from the consultants helping us with data collection. Based on group discussions, a useful strategy can be to have the group members vote on the top 3 behaviors that seem most important to improve (everyone gets to vote for 3 items, then pick the 3 items with the most votes). A participatory process is important to get all group members engaged and committed, and increases the likelihood of behavior change [94, 95].

**Fig. 5:**
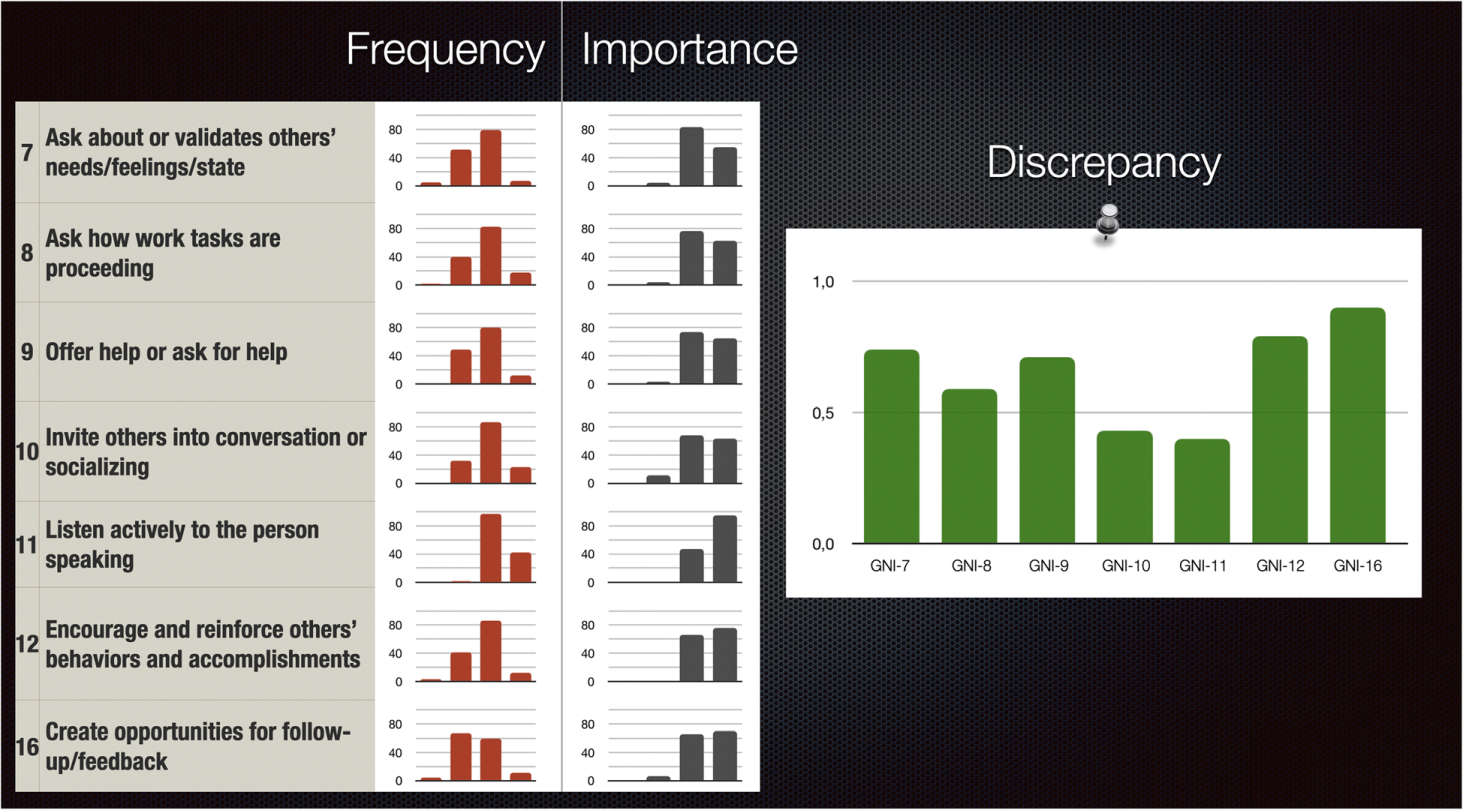
One way to provide graphical GNI feedback for group ratings on items in the Prosocial domain

Providing feedback to a group on their ratings can be done in many ways. Figure 5 shows one way to summarize and visualize frequency and importance ratings, as well as the discrepancy between the two. We also provide a Rmarkdown script to automate the creation of a Powerpoint-like presentation from raw data (see Availability of data and materials).

The importance ratings by themselves are of less interest, at least on the individual level. On a group level, the level of variation or homogeneity in importance ratings within a group could be an interesting variable. The interaction between importance and frequency ratings could be of interest, but it is challenging to find suitable strategies for analysis. The 16 possible combinations of responses (frequency combined with importance) for each item could theoretically be represented with 16 unique numerics and analyzed as categorical variables, but the WLSMV estimator has a maximum of 10 response categories. One strategy could be to classify responders into four groups, using combinations of high (score 3–4) or low (score 1–2) frequency and importance of one or more items, and see how this relates to variables such as stress or social support. However, this kind of dichotomization of data should be done carefully, as it involves discarding a significant amount of variation in the data.

### Limitations

Ideally, more than one data sample would have been available to validate the findings from our analysis, particularly regarding dimensionality. The four CFA models tested were all conducted with the same sample, and we hope that future data collected could be used to make comparative analyses.

What is sorely lacking in this analysis is the validation against real-world objective outcomes [96], such as performance, sick-days, turnover, and economic variables. It is challenging to get access to such data, not least on a group-level, in a sufficient amount for statistical analyses. Also, the correlational analyses of course say nothing about causality. It would be very valuable to study whether changes in the GNI items/behaviors can be found to mediate changes in other constructs in a longitudinal study design. If the behaviors are relevant as targets of change that positively influence the work environment, objective outcomes should also be measurable, and subjective outcomes measured using adequately sensitive instruments, perhaps focused on the predominant models of Effort/Reward Imbalance, and Demands, Control, and Social Support. For instance, a recent meta-analysis [12] showed associations between those two models and sickness absence due to mental health issues. The absence from work due to mental health issues has been rising in many countries [97, 98], and preventive action could perhaps be guided based on GNI assessment.

The Rasch perspective on measurement, particularly regarding targeting and creating items that allow the full range of the construct to be measured, was not used during the item creation stage. For the Non-toxic domain, there is a need to fill some gaps in item thresholds with additional items, as indicated by Fig. 3.

Some items could be optimized if better measurement precision is desired. For example, item 9, “offer help or ask for help”. These are two related but different behaviors and the item could probably be split into two separate items. Another example is item 4, “use discriminatory language/jokes, or laugh at such”, which also consists of two different behaviors. Still, the GNI instrument works reasonably well in its current form, and will likely be helpful in creating opportunities for useful discussions about the items. When a group has agreed on behaviors to improve, they could adapt or create new items that better pinpoint what they are targeting, to measure development over time.

Our sample contains a fairly wide range of organizations and work settings, but the number of participants from each organization was insufficient to analyze differential item functioning (DIF) for the organization variable. Item difficulty may turn out to vary depending on contextual factors relevant to different types of organizations and their work settings. For instance, “invite others into conversation or socializing” might be more challenging to do in a distance work situation compared to a setting where everyone in the group are in the same office space which enables informal and spontaneous conversations. This is extra relevant when many are working from home, but also for those who are road workers or travel extensively. DIF analyses comparing these contextual factors would be very interesting.

Since the number of clusters/groups needed to conduct a multilevel analysis is large, we were unable to provide this type of analysis with the current sample. Hopefully, this study can encourage others to collect data for future group-level analysis. The use of clustering in adjusting for standard errors resulted in an improved model fit, which indicates that there are group-level dependencies in the data.

This analysis only analyzed data using the Swedish language version of GNI. While other translations are available, their measurement properties are unknown.

## Conclusions

We recommend that the GNI primarily be used as an assessment tool for initiating a change process, at least until longitudinal data have been collected to analyze properties such as sensitivity to change and measurement invariance over time. The behaviors that become targets of change based on GNI assessment and feedback sessions should be tracked through ways of measurement established to be reliable, such as observations or ecological momentary assessment. Ideally, such measurements would be conducted in combination with retrospective ratings of the GNI, to further investigate the instruments’ measurement properties in its current form.

An idea for further research on group-level analysis is to investigate whether the level of variation of responses within a group could be an indicator in itself. A large within-group variance could signal that there is a lot of different experiences of what goes on in a group. Depending on the group size, the variation could be clustered around “cliques” of coworkers that work well with each other but not with those in the other clique.

This instrument has “inventory” in its name, and we hope others will add to and/or refine the content of this inventory to improve the assessment properties for various purposes. The basic structure of the questionnaire, assessing both frequency and importance, can hopefully be a good foundation for future development. The GNI is intended primarily as a high utility assessment for groups to guide change and interventions, not as a high precision measurement instrument. It could evolve to also have great precision to reliably track change over time, perhaps both on the individual and group level.

We propose that the concept of nurturance and the behaviors included in the GNI measure are likely to be relevant for other groups and contexts, such as families, couples, and classrooms [99]. These behaviors intend to describe basic social skills that are generally beneficial, no matter the setting. Gathering data from diverse settings would be a very interesting step toward creating a universal assessment of nurturance.

This paper has presented analyses on the individual level that indicates sufficient reliability and validity, and we believe that the GNI can be useful in its current form. We hope that the guidance and materials we have provided in this paper also make the GNI easy to use for anyone interested in assessing and improving work environments.

## Supplementary Information

**Additional file 1 **Table 1**.** Risk factor – Non-toxic (Toxic behaviors reverse scored, higher scores = lower frequency of toxic behaviors). Table 2**.** Protective factors – Prosocial, Limit Problems, and Psychological Flexibility. Wright map illustrating item response thresholds on the same logit scale as person locations for the Non-toxic factor. Wright map illustrating item response thresholds on the same logit scale as person locations for the factor merging all items from Prosocial behaviors, Limit problems, and Psychological Flexibility.

## Data Availability

GNI-23 questionnaires in English, Swedish, and Norwegian are available at 10.17605/OSF.IO/MBT9G Additional file 1 with Rasch score transformation tables is available at 10.17605/OSF.IO/MBT9G. Rmarkdown script for creating a HTML presentation file from data to use with groups when giving feedback is available at 10.17605/OSF.IO/MBT9G The dataset supporting the conclusions of this article is available in the Figshare repository, 10.6084/m9.figshare.13042010

## Abbreviations

GNI: Group Nurturance Inventory
NE: Nurturing Environments
PF: Psychological Flexibility
WAAQ: Work Acceptance and Action Questionnaire
ERI: Effort/Reward Imbalance
SRMR: Standardized Root Mean square Residual
RMSEA: Root Mean Square Error of Approximation
CFI: Bentler’s Comparative Fit Index
TLI: Tucker-Lewis Index
WLSMV: A robust weighted least squares estimator using a diagonal weight matrix
FDI: Factor Determinacy Index
EFA: Exploratory Factor Analysis
CI: Confidence Interval
DCSQ: Demands, Control, and Social Support Questionnaire
PSS: Perceived Stress Scale
SNAQ: Short Negative Acts Questionnaire
PSI: Person Separation Index
SD: Standard Deviation
