# Supplementary material for: The Group Nurturance Inventory — initial psychometric evaluation using Rasch and factor analysis
Source: BMC Public Health. 2021 Jul 26;21:1454. doi: 10.1186/s12889-021-11474-5 (PMC8311413; doi:10.1186/s12889-021-11474-5)
Supplement: Supplementary file 1 — Additional file 1 Table 1. Risk factor – Non-toxic (Toxic behaviors reverse scored, higher scores = lower frequency of toxic behaviors). Table 2. Protective factors – Prosocial, Limit Problems, and Psychological Flexibility. Wright map illustrating item response thresholds on the same logit scale as person locations for the Non-toxic factor. Wright map illustrating item response thresholds on the same logit scale as person locations for the factor merging all items from Prosocial behaviors, Limit problems, and Psychological Flexibility. [file 12889_2021_11474_MOESM1_ESM.docx]

**Appendix**

**Rasch score transformation tables**

Converts raw data sum scores for GNI ratings from ordinal data to an interval scale with measurement uncertainties specified at each level. S.E. = Standard Error.

**Table 1.** Risk factor – Non-toxic (Toxic behaviors reverse scored, higher scores = lower frequency of toxic behaviors)

| Ordinal score | Interval score | S.E. |
| --- | --- | --- |
| 7 | 7.00 | 2.71 |
| 8 | 8.95 | 1.59 |
| 9 | 10.28 | 1.23 |
| 10 | 11.22 | 1.10 |
| 11 | 12.01 | 1.04 |
| 12 | 12.73 | 1.01 |
| 13 | 13.43 | 1.00 |
| 14 | 14.12 | 0.99 |
| 15 | 14.81 | 1.00 |
| 16 | 15.51 | 1.00 |
| 17 | 16.22 | 1.02 |
| 18 | 16.96 | 1.05 |
| 19 | 17.78 | 1.12 |
| 20 | 18.72 | 1.21 |
| 21 | 19.80 | 1.27 |
| 22 | 20.91 | 1.24 |
| 23 | 21.92 | 1.17 |
| 24 | 22.83 | 1.13 |
| 25 | 23.72 | 1.15 |
| 26 | 24.71 | 1.25 |
| 27 | 26.05 | 1.59 |
| 28 | 28.00 | 2.70 |

**Table 2.** Protective factors – Prosocial, Limit Problems, and Psychological Flexibility

| Ordinal score | Interval score | S.E. |
| --- | --- | --- |
| 16 | 16.00 | 5.79 |
| 17 | 20.00 | 3.29 |
| 18 | 22.51 | 2.44 |
| 19 | 24.11 | 2.08 |
| 20 | 25.35 | 1.87 |
| 21 | 26.39 | 1.74 |
| 22 | 27.31 | 1.65 |
| 23 | 28.15 | 1.59 |
| 24 | 28.93 | 1.54 |
| 25 | 29.68 | 1.51 |
| 26 | 30.40 | 1.49 |
| 27 | 31.10 | 1.48 |
| 28 | 31.80 | 1.47 |
| 29 | 32.48 | 1.46 |
| 30 | 33.16 | 1.46 |
| 31 | 33.84 | 1.45 |
| 32 | 34.52 | 1.45 |
| 33 | 35.19 | 1.45 |
| 34 | 35.87 | 1.45 |
| 35 | 36.53 | 1.44 |
| 36 | 37.20 | 1.44 |
| 37 | 37.86 | 1.43 |
| 38 | 38.52 | 1.43 |
| 39 | 39.17 | 1.43 |
| 40 | 39.82 | 1.43 |
| 41 | 40.48 | 1.43 |
| 42 | 41.14 | 1.44 |
| 43 | 41.81 | 1.45 |
| 44 | 42.48 | 1.46 |
| 45 | 43.17 | 1.47 |
| 46 | 43.86 | 1.48 |
| 47 | 44.57 | 1.49 |
| 48 | 45.28 | 1.50 |
| 49 | 46.01 | 1.50 |
| 50 | 46.73 | 1.51 |
| 51 | 47.46 | 1.51 |
| 52 | 48.19 | 1.51 |
| 53 | 48.93 | 1.52 |
| 54 | 49.67 | 1.52 |
| 55 | 50.42 | 1.54 |
| 56 | 51.19 | 1.56 |
| 57 | 51.99 | 1.60 |
| 58 | 52.83 | 1.66 |
| 59 | 53.75 | 1.74 |
| 60 | 54.78 | 1.86 |
| 61 | 56.00 | 2.06 |
| 62 | 57.58 | 2.41 |
| 63 | 60.04 | 3.26 |
| 64 | 64.00 | 5.78 |

**Wright map illustrating item response thresholds on the same logit scale as person locations for the Non-toxic factor**


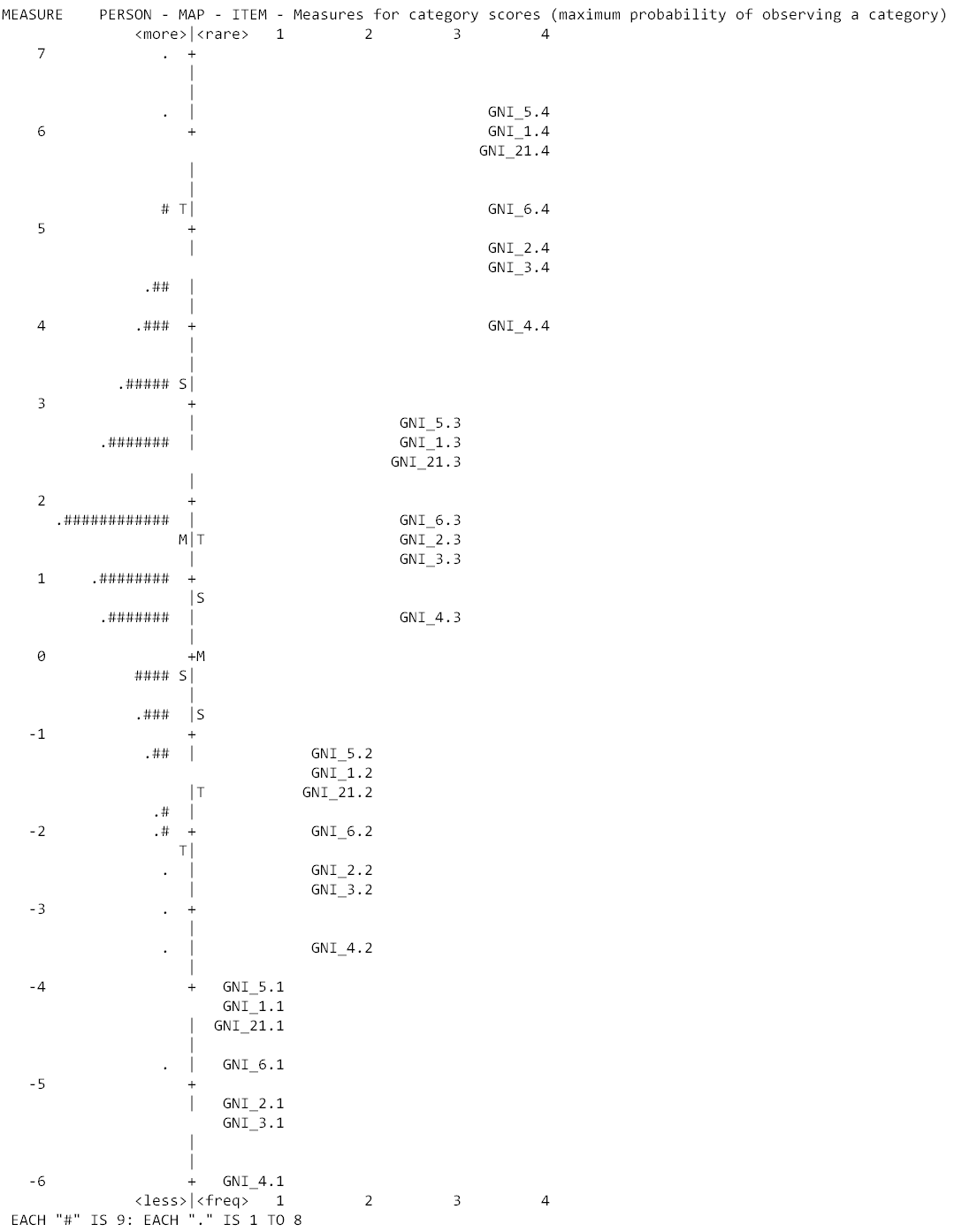


**Wright map illustrating item response thresholds on the same logit scale as person locations for the factor merging all items from Prosocial behaviors, Limit problems, and Psychological Flexibility**

**
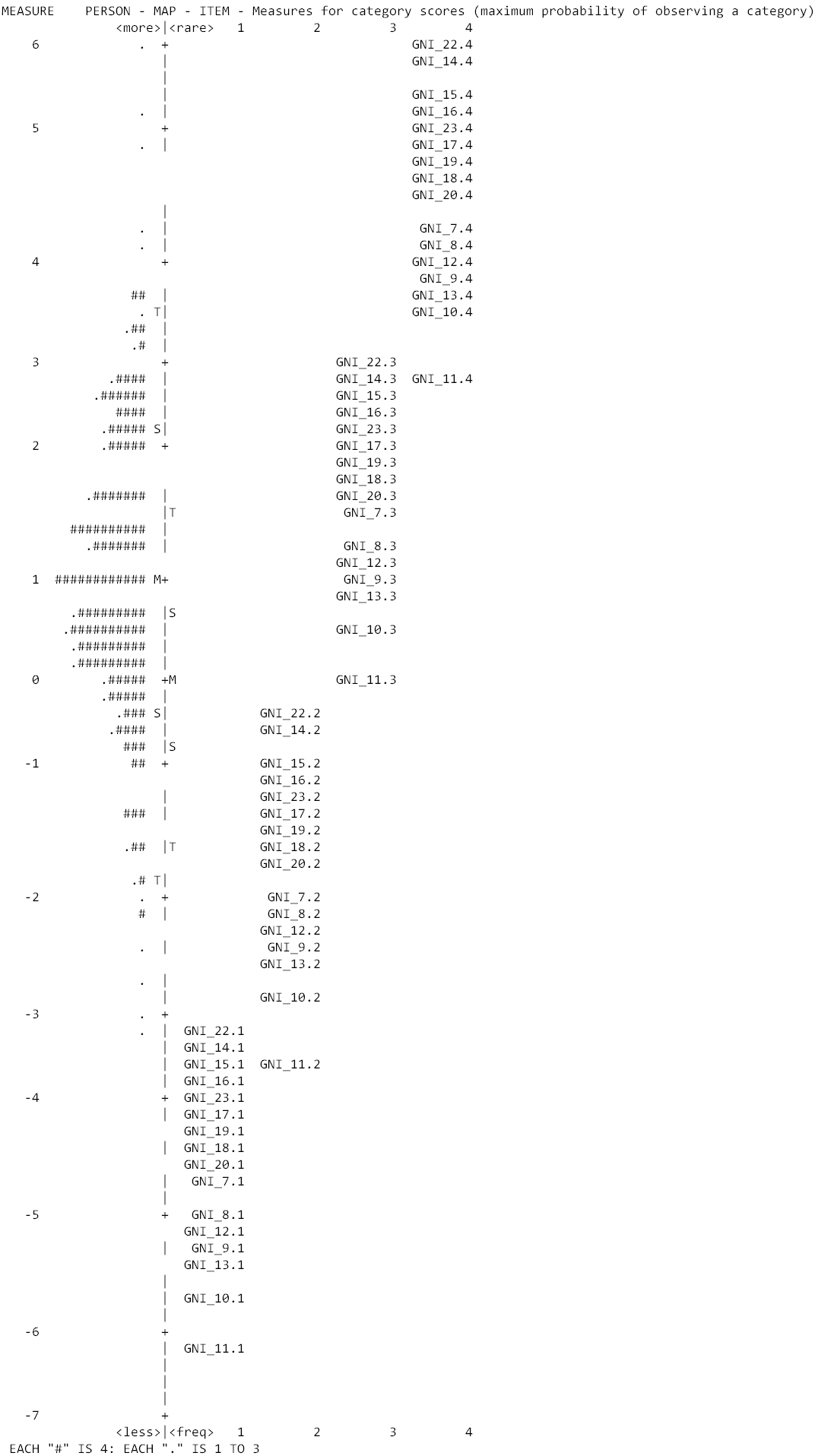
**
